# Influence of the Use of Milk Replacers and pH on the Texture Profiles of Raw and Cooked Meat of Suckling Kids

**DOI:** 10.3390/foods8110589

**Published:** 2019-11-19

**Authors:** Guillermo Ripoll, María J. Alcalde, María G. Córdoba, Rocío Casquete, Anastasio Argüello, Santiago Ruiz-Moyano, Begoña Panea

**Affiliations:** 1 Instituto Agroalimentario de Aragón, IA2, CITA-Universidad de Zaragoza, C/Miguel Servet, 177, 50013 Zaragoza, Spain; bpanea@aragon.es; 2 Centro de Investigación y Tecnología Agroalimentaria de Aragón, Avda. Montañana, 930, 50059 Zaragoza, Spain; 3 Department of Agroforestry Science. Universidad de Sevilla. Crta. Utrera, 41013 Sevilla, Spain; aldea@us.es; 4 Instituto Universitario de Investigación de Recursos Agrarios (INURA), Nutrición y Bromatología, Escuela de Ingeniería Agrarias, Universidad de Extremadura, Avda. Adolfo Suarez s/n, 06007 Badajoz, Spain; mdeguia@unex.es (M.G.C.); rociocp@unex.es (R.C.); srmsh@unex.es (S.R.-M.); 5 Department of Animal Pathology, Animal Production and Science and Technology of Foods, Universidad de Las Palmas de Gran Canaria, 35416 Las Palmas, Spain; tacho@ulpgc.es

**Keywords:** rearing system, stress, DFD, TPA, hardness, toughness, shear force, Warner-Bratzler

## Abstract

The aim of this work was to study the texture profile of fresh and cooked *longissimus* *thoracis et lumborum* muscle from suckling kids raised with natural milk or milk replacers. Suckling male kids from eight goat breeds (Florida, FL; Cabra del Guadarrama, GU; Majorera, MA; Palmera, PL; Payoya, PY; Retinta, RE; Tinerfeña, TI; Verata, VE), all of single parturition, were raised with milk replacers (MR) or with natural milk from the dams (NM). The meat pH, Warner-Bratzler shear force, texture profile analysis and chemical composition were determined. Kids were clustered based on their pH by k-means clustering. The effect of the rearing system on the textural profile was strongly modulated by breed. The values of Warner-Bratzler shear force and hardness found in these breeds under both rearing systems were very low. Hence, the toughness of very light suckling kids should not be a determining factor in choosing a breed or rearing system. Nevertheless, the use of milk replacers increased the presence of meat with high pH, which modified the textural parameters, decreasing the shear force but increasing cohesiveness and adhesiveness. Consequently, depending on the commercial strategy of the farm, the election of the breed and rearing system must be considered together.

## 1. Introduction

Approximately 4.7 million head of goats and kids were slaughtered within the European Union in 2017 [1]). Meat from goats is considered healthy because it is low in calories and fat [2]. However, Mediterranean goat farms are mainly focused on production of cheese and milk products because they have higher prices than cow milk [3,4,5]. When kid goats are reared with their dams, the availability of milk for cheese production is decreased, and the quality of milk may change. Although most of the incomes per goat on the dairy farm come from the sale of milk, 20% of the total income comes from the sale of kids [6]. These kids are weaned very early and reared with milk replacers. Milk replacers specifically formulated for kids result in good daily weight gain. The kids are mostly slaughtered at a very light carcass weight of 5–7 kg, and this meat is perceived by consumers to be of high quality [7]. However, some farmers believe that kids reared with milk replacers provide tougher meat [8] and are opposed to this practice. This belief could be explained by the fact that most of the kid meat with high pH comes from kids raised on milk replacers [9], which might induce tough meat. On the other hand, meat of kids reared with milk replacers was preferred by consumers based on its appearance. Additionally, the purchase intentions were greater for kids reared with milk replacers [10].

Meat sensory evaluation is determined from a complex interaction of sensory and physical processes during chewing, with tenderness being the most important [11]. Tenderness is the sensory variable that is most related to the overall appraisal [12]. Therefore, several instrumental methods have been developed to study the textural characteristics of meat. The most important are the Warner-Bratzler method [13], which provides a main variable based on the maximum force to shear the sample and is usually used with cooked meat; few studies have used the Warner-Bratzler method to assess raw meat. The texture profile analysis (TPA) is also a widely used method. This test provides a set of variables describing the rheological characteristics of meat and has been used in both raw and cooked meat. Both instrumental methods are often used as an approximation of sensory tenderness because they are easier and cheaper than sensory analysis. The use of raw meat is quick, but it is cooked meat that is consumed by people. TPA of raw meat predicted sensory tenderness better than the Warner-Bratzler method, but the Warner-Bratzler method was more correlated with the sensory tenderness of cooked meat than the TPA [14]. Therefore, it seems that the best options to analyze meat tenderness are the TPA for raw meat and the Warner-Bratzler method for cooked meat. There have been some studies about the Warner-Bratzler shear force of suckling kids [7,8,15,16,17,18,19], but there have been no such studies using TPA. Because pH and milk quality could affect kid meat texture and because information about TPA of suckling kid meat is scarce, the aim of this work was to study the texture profile of fresh and cooked meat from suckling kids raised with natural milk or milk replacers.

## 2. Materials and Methods

### 2.1. Animals

All procedures were conducted according to the guidelines of Directive 2010/63/EU on the protection of animals used for experimental and other scientific purposes [20]. Suckling male kids of eight goat breeds (Florida, FL; Cabra del Guadarrama, GU; Majorera, MA; Palmera, PL; Payoya, PY; Retinta, RE; Tinerfeña, TI; Verata, VE; US) were reared on two or three farms per breed in their respective local areas. Animals were all born from single parturition, and half were raised with milk replacers (MR), while the other half were raised with natural milk from the dams (NM). Kids in the MR rearing system were fed colostrum for the first 2 days and had free access to milk replacer 24 h a day, which was suckled from a teat connected to a unit for feeding a liquid diet. Commercial milk replacers were reconstituted at 17% (w/v) and given warm (40 °C). The main ingredients were skimmed milk (≈60%) and whey. The chemical composition of the milk replacers was as follows: total fat 25% ± 0.6, crude protein 24% ± 0.5, crude cellulose 0.1% ± 0.0, ash 7% ± 0.6, Ca 0.8% ± 0.1, Na 0.5% ± 0.2, P 0.7% ± 0.0, Fe 36 mg/kg ± 4.0, Cu 3 mg/kg ± 1.7, Zn 52 mg/kg ± 18.8, Mn 42 mg/kg ± 14.4, I 0.22 mg/kg ± 0.06, Se 0.1 mg/kg ± 0.06 and BHT 65 ppm ± 30. Kids in the NM rearing system were kept separated from their dams during the day while the dams grazed. At night, they were housed with their dams in a stable and suckled directly from dams with no additional feedstuff. Kids from both rearing systems had no access to concentrates, hay, forages or other supplements. The natural milk of goats at 30 d *postpartum* was collected in the morning, and the chemical composition of the milk was determined using a DMA2001 milk analyzer (Miris Inc., Uppsala, Sweden).

### 2.2. Carcass Sampling

The numbers of kids used are shown in Table 1. The 246 kids were slaughtered at a live weight of 8.47 kg ± 0.077 kg. Because the kids of the different breeds were raised in different places, to minimize the effect of the transport, they were slaughtered in a slaughterhouse close to each farm, hence, the duration of the transport from farm to slaughterhouse ranged from 30 to 60 min. The animals of different groups and farms were never mixed during transport or at the slaughterhouse. Standard commercial procedures according to the European normative for protection of animals at the time of killing [21] were followed. Head-only electrical stunning was applied (1.00 A) to kids, which were then exsanguinated and dressed. Thereafter, the hot carcasses, including head and kidneys, were weighed to achieve a hot carcass weight (HCW) of 4.97 kg ± 0.061 kg. Afterwards, the carcasses were hung by the Achilles tendon and chilled for 24 h at 4 °C.

After carcass chilling, the *longissimus thoracis et lumborum* muscle of both the left and right halves of the carcasses were extracted and sliced. The pH was measured on the left *longissimus thoracis* with a pH-meter equipped with a Crison 507 penetrating electrode (Crison Instruments S.A., Barcelona, Spain). Then, the left *longissimus thoracis* was vacuum packed and frozen at −20 °C until chemical composition analyses. The right *longissimus thoracis* was vacuum packed, aged for 3 days at 4 °C in darkness and frozen at −20 °C until Warner-Bratzler maximum stress determination. The *longissimus lumborum* muscle of both the left and right half carcasses were extracted, vacuum packed, aged for 3 days and frozen at −20 °C until TPA of raw and cooked meat, respectively.

### 2.3. Meat Chemical Composition

The moisture content of meat (Moist) was determined by dehydration at 100 °C to a constant weight by the ISO recommended methods (ISO, 1973). Crude protein (CP) was determined following the Kjeldahl method [22]. Intramuscular fat content (IMF) was quantified using the method of Bligh and Dyer [23]. Ash content was assessed by dividing the weight before and after ignition in a muffle furnace for 8 h [22] (Ash). Analyses were run in duplicate and expressed as the percentage of fresh meat. Non-protein nitrogen (NPN) was determined by the Nessler method using 4 g of sample after protein precipitation with 0.6 M perchloric acid, and amino acid nitrogen (AN) was determined from the 0.6 M perchloric acid protein precipitation fraction after peptide precipitation with 10% sulfosalicylic acid as described in Benito, et al. [24].

### 2.4. Meat Texture

An Instron machine model 5543 (Instron Limited, Cerdanyola, Spain) was used to determine the shear force of cooked *longissimus thoracis* (LT). Samples were thawed in tap water for 4 h until they reached an internal temperature of 16–19 °C. Then, the samples were heated in a water bath at 75 °C to an internal temperature of 70 °C. Temperature was controlled with a Testo 108-2 waterproof food thermometer with a Type T thermocouple (Instrumentos Testo S.A., Cabrils, Spain). Then, the steaks were cooled overnight at room temperature. Cross-sectioned meat blocks of 1 cm^2^ and a 3 cm length were measured with a Mitutoyo digital caliper (Mitutoyo Co., Kawasaky, Japan) with a resolution of 0.01 mm. The samples were sheared perpendicularly to the long axis of the block using a Warner-Bratzler device with a cross-head speed of 2.5 mm s^−1^. The maximum stress (load at maximum peak shear force per unit of cross-section, in N cm^−2^) was determined.

Samples of *longissimus lumborum* (LL) were thawed in tap water for 4 h until they reached an internal temperature of 16–19 °C. Then, samples of the left LL were heated in a water bath at 75 °C to an internal temperature of 70 °C; samples of the right LL remained raw. TPA was performed at room temperature using a TA.XTA2i texture analyzer (Stable Micro Systems, Godalming, UK). One cylinder with a 1.5 cm height and 2 cm diameter was prepared from every sample. A double compression cycle test was performed at up to 50% compression of the original portion height with an aluminum cylinder probe with a 6 cm diameter. A time of 5 s was allowed to elapse between the two compression cycles. Force–time deformation curves were obtained with a 250 N load cell applied at a cross-head speed of 1 mm/s. The following parameters were quantified: hardness (maximum force of the first compression cycle required to compress the sample, N), adhesiveness (negative area under the abscissa after the first compression, N·s), springiness (ability of the sample to recover its original form after the deforming force was removed, cm), cohesiveness (extent to which the sample could be deformed prior to rupture, dimensionless), chewiness (work required to masticate a solid food before swallowing, J) and resilience (ability of a product to recover its original height, dimensionless).

### 2.5. Statistical Analysis

All statistics were calculated using the XLSTAT statistical package v.3.05 (Addinsoft, New York, NY, USA). Studied variables were analyzed using the ANCOVA procedure with the breed (B) and the rearing system (RS) as fixed effects and the hot carcass weight (HCW) as a covariate. Least square means were adjusted for an HCW of 4.965 kg, and differences were tested with the Bonferroni test at a 0.05 level of significance. Pearson’s correlations between the raw studied variables and between the residuals of variables were calculated. Principal component analysis (PCA) was performed by projecting the pH, chemical composition and textural variables as active variables and the rearing system and breed as supplementary data to highlight the associations between the loadings of the variables [25]. There was used the varimax rotation and a biplot of variables and centroids were plotted. Kids were clustered together based on their pH by k-means clustering using Wilk’s lambda as classification criterion. The statistical procedure tested the classification from 2 to 5 clusters to maximize the intergroup variability and minimize the intragroup variability. The number of clusters was selected to ensure significant pH differences among all clusters and to avoid clusters formed by 10 or fewer observations. The inter- and intra-class variabilities of the clusters were 80.8% and 19.2%. Then, ANCOVA was carried out for the pH and texture variables, with the pH cluster as a fixed effect and HCW as a covariate. A Duncan test was used to compare means, with a significance of *p* < 0.05. The independence between the rearing system and the pH clusters was tested with the χ^2^ test.

## 3. Results

### 3.1. Chemical Composition of Natural Milk and Longissimus Thoracis Muscle

The chemical composition of the natural milk of goats is shown in Table 1. Differences between breeds were found in protein and fat percentages (*p* < 0.001). Majorera, Palmera and Tinerfeña had the highest values of protein, while Cabra del Guadarrama had the lowest such value. In addition, Cabra del Guadarrama had the lowest value of fat, and Verata had the highest value. The lactose percentages ranged from 4.05 to 4.25 without differences among breeds (*p* > 0.05). 

The chemical composition of longissimus thoracis muscle is shown in Table 2. The pH at 24 h ranged from 5.53 to 6.16. There was a significant interaction between breed and rearing system (*p* < 0.001). Most of the breeds had the same pH when the kids were reared in both rearing systems. However, the Payoya and Tinerfeña kids reared with milk replacers had greater pH than those kids reared with natural milk (*p* < 0.05). The rearing system did not affect the percentage of intramuscular fat (IMF), whereas breed did (*p* < 0.001). Guadarrama kids had the greatest values of IMF, and Florida, Majorera, Palmera, Payoya and Tinerfeña kids had the lowest (*p* < 0.05), while Retinta and Verata kids had intermediate values (*p* < 0.05). The protein percentage was affected by an interaction between breed and rearing system (*p* < 0.001). Therefore, kids of Guadarrama, Majorera and Payoya fed natural milk (NM) had a greater percentage of protein than kids fed milk replacers (MR) (*p* < 0.05). However, the kids of the other breeds had a similar percentage of protein in both rearing systems (*p* > 0.05).

### 3.2. Meat Texture 

The texture profile of raw longissimus thoracis muscle is shown in Table 3. The interaction between rearing system and breed was significant (*p* < 0.001) for every variable, but in general, the effect was more noticeable for breed than for rearing system. The Retinta breed was mostly affected by the rearing system, with a higher hardness in the milk replacer system than in the natural system, whereas adhesiveness, cohesiveness and resilience presented higher values for the natural milk system. In the Majorera breed, the rearing system affected only springiness and resilience, both of which were higher in the natural milk system than in the milk replacer system. In contrast, in the Verata breed, springiness was higher in the natural milk system than in the milk replacer system, with the rest of the variables being unaffected. Finally, in the Payoya breed, the only variable affected by the rearing system was chewiness, which was higher in the natural milk system than in the milk replacer system. The Florida, Cabra del Guadarrama, Palmera and Tinerfeña breeds were not affected at all (*p* < 0.05).

Regarding the breed effect on the raw texture profile, the Retinta breed presented higher values for adhesiveness, and Verata had the highest values for chewiness, independent of the rearing system (*p* < 0.05). In addition, in the milk replacer system, Retinta presented the lowest values for cohesiveness and resilience, whereas Cabra del Guadarrama presented the lowest chewiness values, and Palmera presented the highest values for resilience. In the natural milk system, Payoya presented higher values for chewiness than the other breeds (*p* < 0.05). Chewiness was affected only by breed (*p* < 0.001).

The texture profile of cooked longissimus thoracis muscle is shown in Table 4. The interaction between rearing system and breed was significant (*p* < 0.001) for every variable except chewiness (*p* > 0.05). The rearing system affected the hardness, adhesiveness, springiness and cohesiveness only of Retinta kids. The use of milk replacers increased the two first variables and decreased the two latter variables (*p* < 0.05). There was an effect of breed but not rearing system on chewiness (*p* < 0.001). The resilience of Florida and Retinta was increased and decreased, respectively, by the use of milk replacers (*p* < 0.05). Cooking had a great effect, decreasing hardness and adhesiveness while decreasing cohesiveness. Chewiness and resilience were slightly affected by cooking, and springiness was not affected.

**Figure 1:**
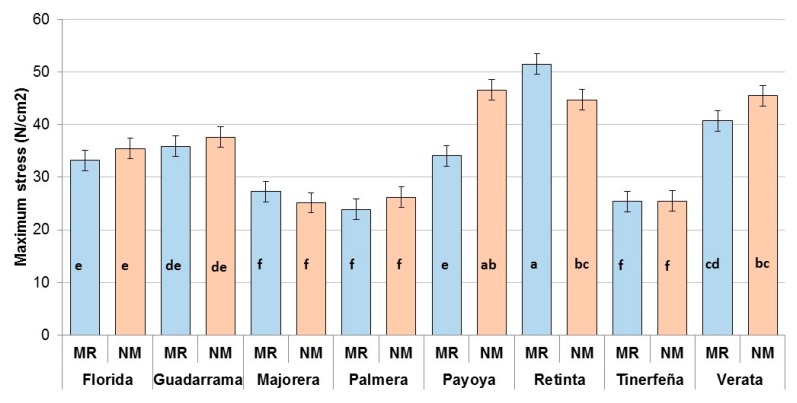
Warner-Bratzler maximum stress of *longissimus thoracis* muscle from kids reared with milk replacer (**MR**) or natural milk from their dams (**NM**). Different superscripts indicate significant differences (*p < 0.05*).

The Warner-Bratzler maximum stress is shown in Figure 1. The maximum stress was affected by the significant interaction (*p* = 0.0002) between the breed and rearing system. In addition, the covariate of HCW was also significant (*p* = 0.004). The maximum stress of Payoya and Retinta were affected by the rearing system (*p* < 0.05) but in opposite ways. Florida, Guadarrama, Payoya, Retinta and Verata had maximum stress values greater than 30 N cm−2, while Majorera, Palmera and Tinerfeña had lower values (*p* < 0.05).

### 3.3. Principal Component Analysis and Correlations 

There were many significant correlations among the studied variables. Warner-Bratzler maximum stress was significantly correlated with the TPA results of raw and cooked meat (*p* < 0.001), except for chewiness (raw) and resilience (cooked) (*p* < 0.05). However, most of the correlations became nonsignificant (*p* > 0.05) when correlation analysis was performed with residuals of variables. This general absence of correlations demonstrates that there was an important influence of carcass weight. Hence, the Warner-Bratzler maximum stress *was not* correlated with any of the variables (*p* > 0.05). Similarly, there was no correlation between the chemical composition of natural milk and muscle (*p* > 0.05) or between the chemical composition of muscle and the TPA results of cooked meat (*p* > 0.05). However, moisture was correlated with the cohesiveness (0.14; *p* < 0.01) and chewiness (0.14; *p* < 0.01) of raw meat, and protein percentage was correlated with resilience (0.18; *p* = 0.006). Raw meat TPA variables were correlated among themselves (from −0.17; *p* = 0.008 to 0.91; *p* < 0.001) similarly to cooked meat TPA variables (from 0.23; *p* < 0.001 to 0.90; *p* < 0.001). Correlations between the TPA variables of raw and cooked meat were small but significant (from 0.13; *p* = 0.04 to 0.27; *p* < 0.001).

**Figure 2:**
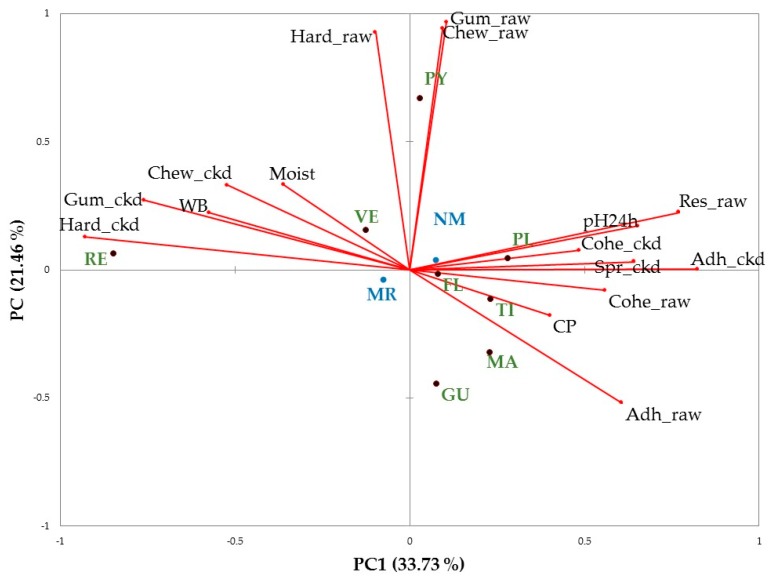
Bi-plot of the texture variables. MR, milk replacer; NM, natural milk; raw, variables determined on raw meat; ckd, variables determined on cooked meat; Hard, hardness; Adh, adhesiveness; Spr, springiness; Cohe, cohesiveness; Chew, chewiness; Res, resilience; WB, Warner-Bratzler maximum stress; Moist, moisture; CP, crude protein; FL, Florida; GU, del Guadarrama; MA, Majorera; PL, Palmera; PY, Payoya; RE, Retinta; TI, Tinerfeña; VE, Verata. The overall Kaiser-Mayer-Olkin score of the PCA was 0.73 (Bartlett’s test of sphericity was significant, *p* < 0.001). The eigenvalues of PC 1 and PC2 are 5.6 and 3.2, respectively.

A principal component analysis were made with the chemical composition, TPA on raw and cooked meat and Warner-Bratzler maximum stress. There were five principal components with eigenvalues higher than 1 explaining the 81.7% of variability. Figure 2 shows the bi-plot of the two first principal components, explaining the 55.2% of the variability. 

Resilience of cooked meat, springiness of raw meat, amino acidic nitrogen, non-protein nitrogen, ash and intramuscular fat were not included in the final PCA due to their Kaiser-Meyer-Olkin values. Three breeds (TI, PL and FL) were placed closer to the abscissas axis in the positive side, being related with cohesiveness on both raw and cooked meat, springiness and adhesiveness on cooked meat, resilience on raw meat and pH. The natural milk rearing system was also related with those variables but it had less importance than breeds. In the opposite side of the abscissas axis were placed moisture and chewiness, gumminess, hardness and Warner-Bratzler maximum stress on cooked meat. RE was the breed related with these variables while VE was placed close to the origin of coordinates. Chewiness, gumminess and hardness on raw meat were related positively to the second PC and Payoya was placed together with those variables. Finally, GU and MA were related with adhesiveness on raw meat. Although most of the studied variables were affected significantly by an interaction between rearing system and breed, according the PCA the effect of breed was more important than the effect of rearing system.

### 3.4. Effect of PH on Kids Meat Quality

Once the meat samples were clustered according their pH at 24 h, the percentage of samples within each cluster, average pH and percentage of kids in the MR group within each cluster were calculated and are shown in Table 5. There were significant differences in pH between clusters (*p* < 0.001). There was also a relationship between the rearing system and the pH cluster (χ^2^ = 13.8; *p* = 0.001). Therefore, the frequency of kids from both rearing systems was similar in the first and second clusters, but 76.7% of kids from cluster 3 with an average pH of 6.2 were in the MR group.

## 4. Discussion

On average, the goat’s milk in this study had a higher content of fat and protein than those reported by other authors using comparable breeds [26,27,28,29,30,31,32]. However, the milk had similar lactose levels to those reported by several authors [26,28,30], but lower levels than those reported by others [29,31,33]. Both the fat and protein content are correlated with the energy of the diet, although in an opposite way. Undernutrition mainly due to grazing results in a decrease in protein and an increase in fat due to the mobilization of body fat [26]. However, the high protein content in this study demonstrates that the goats were fed adequately.

High pH values for kid meat are widespread in the literature, suggesting that goats are generally highly prone to stress [34,35]. The pH values found in the literature for kids were similar to those reported in the present study [7,9,36,37,38,39,40,41] with comparable farming systems and slaughter weights. Most of the reported values were in the range from 5.5 to 5.8, which is considered optimal for goat meat [42]. However, Tinerfeña raised with natural milk had a pH = 6.01, indicating preslaughtering stress [43]. While suckling lambs reared with natural milk or milk replacers had the same pH [44], kids are very sensitive to preslaughter stress (from transport, lairage, isolation, etc.) [39]. Young kids are more susceptible to emotional stress than old ones [40] because younger animals are still largely dependent on their dams [45]. Therefore, the higher frequency of kids fed milk replacers with high pH values could be explained because kids weaned early do not have enough skills to manage emotional stress [45]. The consequences of preslaughter stress are well known and are often responsible for DFD meats [43]. However, the meat of the group with high pH values did not show a modified moisture content. Moreover, the Warner-Bratzler shear force and the hardness of the cooked meat were lower in the meats with high pH than in the meat with low pH. Watanabe, et al. [46] reported that toughness increased from 5.5 to 5.8 with higher pH values. However, high pH values increased other textural parameters, such as chewiness and adhesiveness. Therefore, high pH kid meat is not tough but may be perceived as different by consumers. In addition to these different textural characteristics, high pH values are undesirable because the spoilage of meat increases when the pH is close to 7 [47].

The chemical composition of light suckling kid meat, especially intramuscular fat, was influenced mainly by the breed, but the rearing system had a slight influence. In agreement with these results, Zurita-Herrera, Delgado, Argüello, Camacho and Germano [18] and Argüello, Castro, Capote and Solomon [8] did not find differences in the chemical composition of LTL between the same rearing systems. It has been confirmed that the low amount of IMF is characteristic of suckling kids [48,49], because visceral fat deposits tend to be increased before intramuscular fat deposits in goats [50]. However, an exception was found in Cabra del Guadarrama with IMF higher than 4 %. 

To the best of our knowledge, there have been no TPA studies of suckling kid meat, either raw or cooked. Nor has there been any TPA study of suckling lambs with comparable slaughter weights. Choi, et al. [51] reported that the meat of Australian lamb had higher hardness and adhesiveness, similar springiness, chewiness and cohesiveness and lower cohesiveness than those of kid meat. Önenç, et al. [52] also reported higher values of hardness but lower chewiness of lamb meat compared to suckling kid meat. Bañón, Vila, Price, Ferrandini and Garrido [36] did not use the TPA but studied the sensory characteristics of suckling kids. Hence, these researchers did not find differences in chewiness between rearing systems, but meat from kids fed milk replacers was tenderer than that from natural milk-fed kids. Comparing more meats to suckling kid meat, duck cooked breast had higher hardness, lower chewiness and springiness and similar cohesiveness and resilience as kid meat [53]. Both raw and cooked chicken breast had higher hardness and lower chewiness than kid meat [54]. Romero de Ávila, et al. [55] performed a TPA of cooked hams, which had higher hardness and lower adhesiveness, cohesiveness and springiness than suckling kid meat. However, different variations in the TPA parameters, such as the compression ratio and speed, and the dimensions of the samples make the comparison of results challenging [56]. Therefore, Wee, et al. [57] measured the texture profile of 59 foods and found significant correlations between the chemical composition and the textural properties of food. These authors reported that carbohydrate content decreases hardness. Adhesiveness was the variable most influenced by chemical composition, being increased by humidity and decreased by protein and fat contents. Regarding the effect of cooking, Ruiz de Huidobro, Miguel, Blazquez and Onega [14] reported that hardness, chewiness and springiness of beef increase with cooking in disagreement with our results.

While TPA data are scarce, there is more information about the application of the Warner-Bratzler method to suckling kids and lambs. The literature often compares the use of natural milk and milk replacers to raise kids of just one breed, so conclusions about the influence of milk replacers on meat quality are misleading. Hence, it has been reported that meat toughness is not affected by the rearing system when suckling lambs and kids are slaughtered at very low live weight [8,44]. This is likely because collagen content and solubility are more affected by the age [47] than by the rearing system [8,18,36]. However, we found that some breeds, such as Payoya and Retinta, were affected by the rearing system but were affected in contrasting ways. Unfortunately, as far as we know, there are no similar studies focused on these breeds. Zurita-Herrera, Delgado, Argüello, Camacho and Germano [18] reported low Warner-Bratzler shear force at 1 d *postmortem* on kids of Murciano-Granadina fed milk replacers. Values of approximately 30 N have been reported for very light suckling kids, such as *capretto* and *cabrito* at 1–2 d *postmortem* [18,19,43], in agreement with the results of our study. Other authors reported higher values than 30 N for kid meat [4,5,58] in meat aged from 1 d to 3 d. These values are also lower than those reported for other meats [59,60,61]. Additionally, these values are lower than the values of extremely tender beef [62,63]. Shackelford, et al. [64] reported that meat having Warner-Bratzler shear force values higher than 54 N would be assessed as tough by consumers. However, the transition from tough to tender occurred between 42 N and 48 N [63]. In our study, cooked meat had slightly less hardness than raw meat. Cooking softens the connective tissue but toughs the myofibrils. Therefore, the meat would be tougher or tenderer depending on the temperature and cooking time [47]. Machlik and Draudt [65] studied the influence of cooking time and temperature in very small cylinders of beef. These authors concluded that heating meat at 71ºC decreased the toughness during the first 9 min of cooking. The samples of the kids were also small, and the samples reached the temperature endpoint quickly. Thus, toughness diminished due to cleavage of the peptide bonds and mature crosslinks [47].

## 5. Conclusions

The effect of rearing system on the textural profile was strongly modulated by breed. The values of Warner-Bratzler shear force and hardness found in these breeds under both rearing systems were very low. Hence, the toughness of very light suckling kids should not be a determining factor in choosing a breed or rearing system. Nevertheless, the use of milk replacers increased the pH of meat, which modified the textural parameters, decreasing the shear force but increasing cohesiveness, adhesiveness and cohesiveness. Consequently, depending on the commercial strategy of the farm, the election of the breed and rearing system must be considered together.

## Figures and Tables

**Table 1:** Means, standard error and *p*-value for breed effect on proximal composition of natural milk 30 d post birth in eight goat breeds.

Breed	Protein, %	Fat, %	Lactose, %
Florida	3.79 ^b^	5.11 ^bc^	4.05
Cabra del Guadarrama	3.35 ^c^	4.09 ^e^	4.10
Majorera	4.55 ^a^	4.78 ^cd^	4.11
Palmera	4.54 ^a^	5.40 ^ab^	4.16
Payoya	3.78 ^b^	4.29 ^de^	4.19
Retinta	3.79 ^b^	5.38 ^ab^	4.20
Tinerfeña	4.30 ^a^	4.58 ^de^	4.10
Verata	4.22 ^ab^	5.82 ^a^	4.25
Standard error	0.147	0.181	0.062
Breed effect (*p-value*)	<0.001	<0.001	0.33

Different superscripts indicate significant differences (*p* < 0.05).

**Table 2:** Value of pH at 24 h and chemical composition of *longissimus thoracis* muscle of kids reared with milk replacer (MR) or natural milk from their dams (NM).

B ^†^	RS	n	pH 24 h	Moist, %	IMF, %	CP, %	Ash, %	NPN, mg/g	AN, mg/g
FL	MR	15	5.69 ^de^	78.03 ^a^	1.88 ^def^	18.30 ^de^	1.18 ^ab^	3.086 ^bcde^	0.822 ^cde^
	NM	15	5.61 ^efg^	77.11 ^ab^	1.97 ^def^	18.68 ^cd^	1.18 ^ab^	3.491 ^bcd^	1.043 ^bcd^
GU	MR	15	5.66 ^ef^	76.88 ^ab^	4.29 ^a^	19.25 ^cd^	1.11 ^abc^	5.454 ^a^	0.236 ^g^
	NM	16	5.67 ^de^	71.36 ^f^	5.16 ^a^	23.99 ^ab^	0.96 ^e^	4.699 ^ab^	0.290 ^fg^
MA	MR	16	5.81 ^c^	74.50 ^d^	1.78 ^def^	23.22 ^b^	1.08 ^bcd^	3.047 ^bcde^	1.619 ^a^
	NM	16	5.86 ^c^	73.16 ^e^	0.89 ^f^	24.97 ^a^	1.10 ^abc^	1.702 ^def^	1.426 ^ab^
PL	MR	15	6.16 ^a^	75.31 ^cd^	1.72 ^def^	24.05 ^ab^	1.16 ^ab^	2.802 ^bcde^	0.826 ^cde^
	NM	16	5.85 ^c^	74.07 ^de^	1.29 ^ef^	23.82 ^ab^	1.01 ^de^	1.743 ^def^	1.422 ^ab^
PY	MR	16	5.80 ^c^	76.88 ^ab^	1.63 ^ef^	16.89 ^e^	1.17 ^ab^	2.244 ^cde^	0.471 ^efg^
	NM	14	5.78 ^cd^	76.90 ^ab^	1.11 ^f^	20.30 ^c^	1.12 ^abc^	2.695 ^cde^	0.635 ^def^
RE	MR	15	5.53 ^g^	78.08 ^a^	2.68 ^bcd^	19.51 ^cd^	1.10 ^abc^	1.466 ^ef^	0.790 ^cde^
	NM	15	5.55 ^fg^	76.17 ^bc^	2.97 ^bc^	19.25 ^cd^	1.21 ^a^	2.210 ^cdef^	1.113 ^bc^
TI	MR	16	6.01 ^b^	75.06 ^cd^	1.32 ^ef^	23.81 ^ab^	1.08 ^bcd^	3.513 ^bc^	0.983 ^cd^
	NM	16	5.88 ^c^	74.66 ^d^	1.45 ^ef^	23.56 ^ab^	1.04 ^cde^	2.602 ^cde^	1.754 ^a^
VE	MR	15	5.84 ^c^	76.99 ^ab^	3.16 ^b^	18.22 ^de^	0.96 ^e^	2.699 ^cde^	0.685 ^de^
	NM	15	5.79 ^cd^	76.19 ^bc^	2.16 ^cde^	19.41 ^cd^	1.09 ^bcd^	0.476 ^f^	0.688 ^de^
	s.e.		0.039	0.441	0.324	0.511	0.031	0.363	0.080
	B		<0.001	<0.001	0.001	<0.001	0.001	<0.001	<0.001
	RS		0.006	<0.001	0.26	<0.001	0.47	0.002	<0.001
	B*RS		<0.001	<0.001	0.11	<0.001	<0.001	<0.001	<0.001

^†^ B, Breed; RS, Rearing system; s.e., standard error; Moist, percentage of moisture on fresh basis; IMF, percentage of intramuscular fat on fresh basis; CP, percentage of crude protein on fresh basis; Ash, percentage of ashes on fresh basis; NPN, non-protein nitrogen in mg/g fresh meat; AN, amino acid nitrogen in mg AN/g fresh meat; FL, Florida; GU, del Guadarrama; MA, Majorera; PL, Palmera; PY, Payoya; RE, Retinta; TI, Tinerfeña; VE, Verata. Least square means has been adjusted for an HCW of 4.965 Kg. Different superscripts indicate significant differences (*p* < 0.05).

**Table 3:** Texture profile of raw *longissimus lumborum* muscle of suckling kids reared with milk replacer (MR) or natural milk from their dams (NM).

B ^†^	RS	Hardness (N)	Adhesiveness (-N·s)	Springiness (cm)	Cohesiveness (-)	Chewiness (J·10^−2^)	Resilience (-)
FL	MR	14.38 ^bcd^	0.26 ^ab^	0.83 ^bcd^	0.45 ^ab^	5.18 ^cd^	0.246 ^ab^
	NM	14.97 ^bcd^	0.21 ^c^	0.87 ^abc^	0.47 ^ab^	6.60 ^bcd^	0.247 ^ab^
GU	MR	10.46 ^d^	0.09 ^c^	0.90 ^ab^	0.44 ^ab^	4.10 ^d^	0.231 ^abc^
	NM	11.97 ^cd^	0.11 ^c^	0.90 ^ab^	0.47 ^ab^	5.08 ^cd^	0.265 ^a^
MA	MR	11.90 ^cd^	0.11 ^c^	0.80 ^cd^	0.44 ^ab^	4.14 ^d^	0.195 ^c^
	NM	14.21 ^cd^	0.17 ^c^	0.89 ^ab^	0.47 ^ab^	5.96 ^bcd^	0.252 ^ab^
PA	MR	17.60 ^bcd^	0.37 ^bc^	0.93 ^a^	0.46 ^ab^	7.44 ^bc^	0.263 ^a^
	NM	14.99 ^bcd^	0.21 ^c^	0.89 ^ab^	0.48 ^a^	6.37 ^bcd^	0.266 ^a^
PY	MR	18.74 ^bc^	0.29 ^bc^	0.86 ^abc^	0.43 ^ab^	6.80 ^bcd^	0.242 ^ab^
	NM	33.41 ^a^	0.34 ^bc^	0.84 ^abcd^	0.41 ^b^	11.35 ^a^	0.240 ^ab^
RE	MR	22.22 ^b^	0.96 ^a^	0.86 ^abc^	0.35 ^c^	6.58 ^bcd^	0.120 ^d^
	NM	13.81 ^cd^	0.53 ^b^	0.91 ^ab^	0.48 ^a^	5.77 ^bcd^	0.210 ^bc^
TI	MR	12.14 ^cd^	0.14 ^c^	0.90 ^ab^	0.45 ^ab^	4.67 ^cd^	0.237 ^ab^
	NM	17.88 ^bcd^	0.26 ^bc^	0.89 ^ab^	0.47 ^ab^	7.28 ^bc^	0.246 ^ab^
VE	MR	16.72 ^bcd^	0.31 ^bc^	0.89 ^ab^	0.46 ^ab^	8.74 ^ab^	0.210 ^bc^
	NM	14.45 ^bcd^	0.21 ^c^	0.78 ^d^	0.47 ^ab^	5.16 ^ab^	0.236 ^abc^
	s.e.	1.662	0.058	0.016	0.013	0.641	0.009
	B	<0.001	<0.001	<0.001	<0.001	<0.001	<0.001
	RS	0.086	0.044	0.773	<0.001	0.023	<0.001
	B*RS	<0.001	<0.001	<0.001	<0.001	<0.001	<0.001

^†^ B, Breed; RS, Rearing system; s.e., standard error; FL, Florida; GU, del Guadarrama; MA, Majorera; PL, Palmera; PY, Payoya; RE, Retinta; TI, Tinerfeña; VE, Verata. Least square means has been adjusted for an HCW of 4.965 Kg. Different superscripts indicate significant differences (*p* < 0.05).

**Table 4:** Texture profile of cooked *longissimus lumborum* muscle of suckling kids reared with milk replacer (MR) or natural milk from their dams (NM).

B	RS ^†^	Hardness (N)	Adhesiveness (-N·s)	Springiness (cm)	Cohesiveness (-)	Chewiness (J·10^−2^)	Resilience (-)
FL	MR	8.17 ^de^	0.002 ^c^	0.83 ^a^	0.77 ^a^	5.42 ^cde^	0.36 ^ab^
	NM	9.08 ^de^	0.008 ^c^	0.85 ^a^	0.75 ^abc^	5.86 ^cde^	0.30 ^c^
GU	MR	8.27 ^de^	0.008 ^c^	0.86 ^a^	0.65 ^d^	4.67 ^e^	0.31 ^bc^
	NM	8.51 ^de^	0.026 ^c^	0.82 ^a^	0.65 ^d^	4.53 ^e^	0.29 ^c^
MA	MR	6.92 ^e^	0.010 ^c^	0.87 ^a^	0.71 ^abcd^	4.36 ^e^	0.33 ^bc^
	NM	7.77 ^de^	0.040 ^bc^	0.88 ^a^	0.73 ^abcd^	5.09 ^de^	0.34 ^abc^
PA	MR	8.13 ^de^	0.034 ^bc^	0.90 ^a^	0.70 ^abcd^	5.13 ^cde^	0.32 ^bc^
	NM	8.77 ^de^	0.035 ^bc^	0.86 ^a^	0.68 ^cd^	5.23 ^cde^	0.32 ^bc^
PY	MR	11.72 ^cd^	0.012 ^c^	0.85 ^a^	0.76 ^ab^	7.55 ^abc^	0.39 ^a^
	NM	10.38 ^cde^	0.012 ^c^	0.85 ^a^	0.74 ^abc^	6.68 ^bcde^	0.34 ^abc^
RE	MR	28.14 ^a^	0.271 ^a^	0.66 ^b^	0.52 ^e^	9.12 ^a^	0.14 ^e^
	NM	17.53 ^b^	0.092 ^b^	0.80 ^a^	0.65 ^d^	8.31 ^ab^	0.23 ^d^
TI	MR	8.23 ^de^	0.016 ^c^	0.92 ^a^	0.70 ^abcd^	5.33 ^cde^	0.33 ^bc^
	NM	9.08 ^de^	0.037 ^bc^	0.89 ^a^	0.69 ^bcd^	5.58 ^cde^	0.30 ^c^
VE	MR	15.01 ^bc^	0.039 ^bc^	0.82 ^a^	0.70 ^abcd^	8.51 ^ab^	0.28 ^c^
	NM	11.96 ^cd^	0.012 ^c^	0.84 ^a^	0.77 ^abcd^	7.54 ^abcd^	0.32 ^bc^
	s.e.	0.919	0.013	0.023	0.016	0.489	0.012
	B	<0.001	<0.001	<0.001	<0.001	<0.001	<0.001
	RS	0.002	0.011	0.365	0.042	0.518	0.859
	B*RS	<0.001	<0.001	0.001	<0.001	0.438	<0.001

^†^ B, Breed; RS, Rearing system; s.e., standard error; FL, Florida; GU, del Guadarrama; MA, Majorera; PL, Palmera; PY, Payoya; RE, Retinta; TI, Tinerfeña; VE, Verata. Least square means has been adjusted for an HCW of 4.965 Kg. Different superscripts indicate significant differences (*p* < 0.05).

**Table 5:** Comparison of meat pH on chemical composition and texture of meat of the three pH clusters identified by the *k*-means algorithm. The percentage of kids per cluster is between brackets.

Variables	CL1 (51.2%)	CL2 (36.6%)	CL3 (12.2%)	s.e.	Sig.
% of MR kids	54.4	40.5	76.7		0.001
pH	5.6 ^c^	5.8 ^b^	6.2 ^a^	0.020	<0.001
**Chemical composition**					
Moisture, %	75.82	75.61	75.38	0.310	0.689
Intramuscular fat, %	2.59 ^a^	1.96 ^b^	2.12 ^ab^	0.196	0.015
Crude protein, %	20.42 ^b^	21.36 ^b^	22.34 ^a^	0.381	0.010
Ash, %	1.11	1.09	1.10	0.017	0.380
NPN, mg/g	3.08 ^a^	2.48 ^b^	2.89 ^ab^	0.223	0.041
AN, mg/g	0.91	0.97	0.85	0.065	0.397
**TPA raw meat**					
Hardness, N	15.47	16.15	17.94	1.023	0.390
Adhesiveness,-N·s	0.38 ^a^	0.20 ^b^	0.30 ^ab^	0.037	<0.001
Springiness, cm	0.88 ^b^	0.86 ^c^	0.91 ^a^	0.009	0.001
Cohesiveness	0.43 ^b^	0.46 ^a^	0.46 ^a^	0.007	0.005
Chewiness, J·10^−2^	5.86 ^b^	6.29 ^b^	7.60 ^a^	0.371	0.029
Resilience	0.21 ^b^	0.24 ^a^	0.26 ^a^	0.006	<0.001
**TPA cooked meat**					
Hardness, N	13.79 ^a^	9.53 ^b^	9.13 ^b^	0.753	<0.001
Adhesiveness,-N·s	0.08 ^a^	0.02 ^b^	0.02 ^b^	0.009	<0.001
Springiness, cm	0.80 ^b^	0.86 ^a^	0.90 ^a^	0.012	<0.001
Cohesiveness	0.66 ^b^	0.72 ^a^	0.72 ^a^	0.010	<0.001
Chewiness, J·10^−2^	6.40	5.99	6.14	0.296	0.466
Resilience	0.26	0.29	0.34	0.047	0.634
**Warner-Bratzler**					
Max. Stress, N/cm^2^	38.4 ^a^	34.0 ^b^	27.0 ^c^	1.284	<0.001

NPN, non-protein nitrogen in mg/g fresh meat; AN, amino acid nitrogen in mg AN/g fresh meat; MR, milk replacer; s.e., standard error; Sig, signification of the effect. Different superscripts in the same row indicate significant differences (*p < 0.05*).
